# Effects on Murine Behavior and Lifespan of Selectively Decreasing Expression of Mutant Huntingtin Allele by Supt4h Knockdown

**DOI:** 10.1371/journal.pgen.1005043

**Published:** 2015-03-11

**Authors:** Hui-Min Cheng, Yijuang Chern, I-Hui Chen, Chia-Rung Liu, Sih-Huei Li, Seung J. Chun, Frank Rigo, C. Frank Bennett, Ning Deng, Yanan Feng, Chyuan-Sheng Lin, Yu-Ting Yan, Stanley N. Cohen, Tzu-Hao Cheng

**Affiliations:** 1 Institute of Biochemistry and Molecular Biology, National Yang-Ming University, Taipei, Taiwan, Republic of China; 2 Institute of Biomedical Sciences, Academia Sinica, Taipei, Taiwan, Republic of China; 3 ISIS Pharmaceuticals, Carlsbad, California, United States of America; 4 Department of Genetics, Stanford University School of Medicine, Stanford, California, United States of America; 5 Department of Pathology and Cell Biology & Herbert Irving Comprehensive Cancer Center, Columbia University, New York, New York, United States of America; 6 Brain Research Center, National Yang-Ming University, Taipei, Taiwan, Republic of China; University of Minnesota, UNITED STATES

## Abstract

Production of protein containing lengthy stretches of polyglutamine encoded by multiple repeats of the trinucleotide CAG is a hallmark of Huntington’s disease (HD) and of a variety of other inherited degenerative neurological and neuromuscular disorders. Earlier work has shown that interference with production of the transcription elongation protein SUPT4H results in decreased cellular capacity to transcribe mutant huntingtin gene (*Htt*) alleles containing long CAG expansions, but has little effect on expression of genes containing short CAG stretches. zQ175 and R6/2 are genetically engineered mouse strains whose genomes contain human *HTT* alleles that include greatly expanded CAG repeats and which are used as animal models for HD. Here we show that reduction of SUPT4H expression in brains of zQ175 mice by intracerebroventricular bolus injection of antisense 2’-*O*-methoxyethyl oligonucleotides (ASOs) directed against *Supt4h*, or in R6/2 mice by deletion of one copy of the *Supt4h* gene, results in a decrease in mRNA and protein encoded specifically by mutant *Htt* alleles. We further show that reduction of SUPT4H in mouse brains is associated with decreased HTT protein aggregation, and in R6/2 mice, also with prolonged lifespan and delay of the motor impairment that normally develops in these animals. Our findings support the view that targeting of SUPT4H function may be useful as a therapeutic countermeasure against HD.

## Author Summary

Huntington’s disease (HD) is an inherited genetic disorder that leads to degeneration of brain cells and consequently to abnormal body movements, decreased mental capacity, and death. It is one of a group of untreatable degenerative neurological and neuromuscular diseases caused by expansion of gene segments containing multiple tandemly arrayed copies of short DNA sequences called trinucleotide repeats (TNRs). We report here that interference with production of a protein, SUPT4H, that is differentially needed for transcription through mutant *Htt* genes containing expanded TNRs reduces synthesis of abnormal *Htt* messenger RNA and protein, decreases HTT aggregates in murine brains, delays the occurrence of pathological features of HD, and prolongs HD mouse lifespan. Our results suggest that targeting of SUPT4H may be of value in the treatment of HD.

## Introduction

Huntington’s disease (HD) is one of a collection of untreatable and devastating neurodegenerative and neuromuscular diseases that result from expansion of segments of trinucleotide repeats (TNRs) present within certain genes [1–3]. Whereas the huntingtin (*HTT*) gene normally includes fewer than 30 repeats of the glutamine-encoding trinucleotide CAG, expansion to 36 or more repeats results in HTT protein containing a long polyglutamine stretch, leading to HTT protein aggregation and non-canonical protein-protein interactions—and ultimately resulting in neuronal cell death [4–7]. Analogous TNR expansions in other genes underlie certain spinocerebellar atrophies, muscular dystrophies, and other polyglutamine (polyQ)-associated disorders [6–8]. Additional diseases are attributable to expansions of other TNRs or to CAG expansions in non-protein-coding regions of other genes [9–12].

Earlier work has shown that the transcription elongation protein SUPT4H (known in yeast as Spt4), which interacts with its partner SUPT5H (in yeast, Spt5) to form a complex that aids RNA polymerase II processivity [13], is selectively needed for transcription through gene segments containing expanded TNRs. Decreased production of SUPT4H or Spt4 in cultured cells impedes transcription through expanded TNRs and reduces synthesis of protein containing lengthy polyQ stretches without significantly affecting the production of mRNA and protein from alleles containing non-expanded TNRs. In yeast cells, null mutation of *spt4* and consequently, reduced transcription through DNA containing lengthy TNRs, can decrease the abundance of and restore functionality to the resulting protein; in mammalian striatal neurons grown in culture, shRNA directed against *Supt4h* reduces the production, aggregation, and toxicity of mutant HTT protein [13].

The investigations reported here were aimed at learning whether interference with the actions of SUPT4H would selectively decrease the production of *Htt* mRNA and protein derived from mutant *Htt* alleles in whole animal murine models of Huntington’s disease, and if so, whether such a decrease would affect the pathological consequences of TNR expansions. Our findings indicate that decrease in SUPT4H production in cerebral cortex neurons by injection of antisense oligonucleotides (ASOs) into the brains of mice expressing a human *HTT* exon containing expanded CAG repeats [14,15] reduces the abundance of mutant *Htt* mRNA and protein, while having little or no effect on expression of the co-existing normal *Htt* allele. We further found that downregulation of mutant HTT by deletion of a single *Supt4h* allele in R6/2 HD mice—which contain a lengthy CAG repeat within a transgenically introduced first exon of the human *HTT* gene [16]—results in delay of the motor function impairment characteristic of these mice and in prolongation of mouse lifespan.

## Results

### Decreased mutant *Htt* gene expression by *Supt4h* ASO in zQ175 HD model mice

The discovery that transcription of genes containing expanded repeats of CAG or other trinucleotides located in either protein-coding or transcribed non-coding regions of genes is selectively reduced by interference with the actions of the transcription elongation protein SUPT4H or its yeast counterpart, Spt4 [13] identifies SUPT4H as a potential target for therapies for genetic disorders associated with TNR expansions. In initial experiments to investigate this prospect, we injected 2’-*O*-methoxyethyl-modified antisense oligonucleotide directed against *Supt4h* mRNA into the brains of zQ175 mice, which have been engineered to carry a human *HTT* gene exon that includes expanded TNRs [14,15]. The genomes of the adult zQ175 HD mice used in these studies contain an endogenous normal murine *Htt* allele in addition to the modified one. The anti-sense oligonucleotide (ASO) used was shown in preliminary studies to result in ~80% reduction of *Supt4h* mRNA in the mouse endothelioma cell line bEnd.3 cells (ATCC CRL-2299). The procedures we employed (Materials and Methods) have been used previously to correct a splicing abnormality in the *SMN2* gene in transgenic mice [17], and were also shown to reduce HTT protein production from both *Htt* alleles in R6/2, BACHD and YAC128 mice using ASOs directed against the *Htt* gene [18].

**Fig 1:**
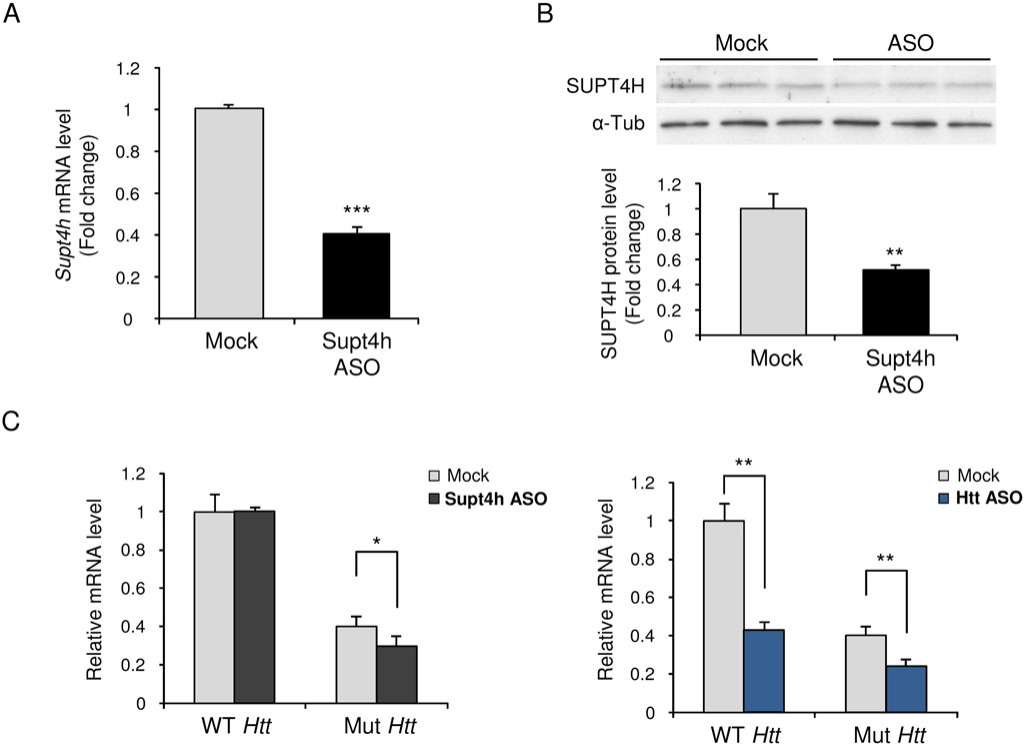
Effect of down regulation by Supt4h ASO on expression of mutant and wild-type *Htt* alleles in zQ175 HD mice. Supt4h ASO was delivered to the brain of zQ175 HD mice by intracerebroventricular (ICV) bolus injection. ASO became distributed throughout CNS via cerebral spinal fluid circulation, and as observed previously [18] the spinal cord most susceptible to its effects. Mice were sacrificed 4 weeks after a single injection at the age of 5.5 months and spinal cords were collected for analyses of ASO effects. (A) *Supt4h* transcript abundance was assessed by quantitative RT-PCR. mRNA level in tissue obtained from PBS-treated zQ175 mice (mock) was set to 1, and relative *Supt4h* mRNA level in tissue from ASO-treated animals is shown. (B) SUPT4H protein level in tissue analyzed in (A) for mRNA abundance was examined by Western blot analysis. After normalization using α-Tubulin, the protein level was compared to mock control. (C) Left, wild-type (WT) and mutant (Mut) *Htt* gene expression were assessed by qRT-PCR in Supt4h ASO-treated samples and compared to that of mock samples. The level of WT *Htt* mRNA in mock samples was set as 1, and *Htt* transcripts produced from the co-existing Mut allele were approximately 40% of WT mRNA obtained from zQ175 KI mice. Right, production of wild type and mutant *Htt* mRNAs following intracerebroventricular bolus injection of an ASO [18] that targets both the WT and Mut alleles of *Htt*. The conditions used for injection and analysis in these experiments were identical for those employed for the ASO targeting *Supt4h* (n = 3 in each group; *, *p* <0.05; **, *p* < 0.01; ***, *p* <0.001 by Student’s *t* test).

Analysis of extracts of entire cerebral cortices (S1 Fig) or lumbar spinal cords collected from mice receiving ASO directed against *Supt4h* showed reduction of *Supt4h* mRNA and protein to 40% or 50% of normal (Fig 1A, 1B). This decrease was accompanied by an approximately 30% decrease from the baseline abundance in untreated zQ175 mouse brains of mutant *Htt* mRNA and protein, which were produced in lesser amounts than *Htt* mRNA and protein from genes containing unexpanded TNRs—as has been reported previously [13,19]. However, injection of ASO directed against *Supt4h* did not result in a detectable change in expression of the *Htt* allele containing an unexpanded TNR, in contrast to the decreased expression in both *Htt* alleles that resulted from injection under identical conditions of an ASO directed against an *Htt* gene sequence (Fig 1C and S2 Fig). The selective decrease in mutant *Htt* expression observed during ASO-mediated targeting of *Supt4h* in mouse brain and spinal cord tissues contrasts with the non-selectively decreased expression of *Htt* resulting from similarly performed intracerebroventricular bolus injection of ASO directed against *Htt* mRNA [18].

### Generation of mice lacking an *Supt4h* allele

**Fig 2:**
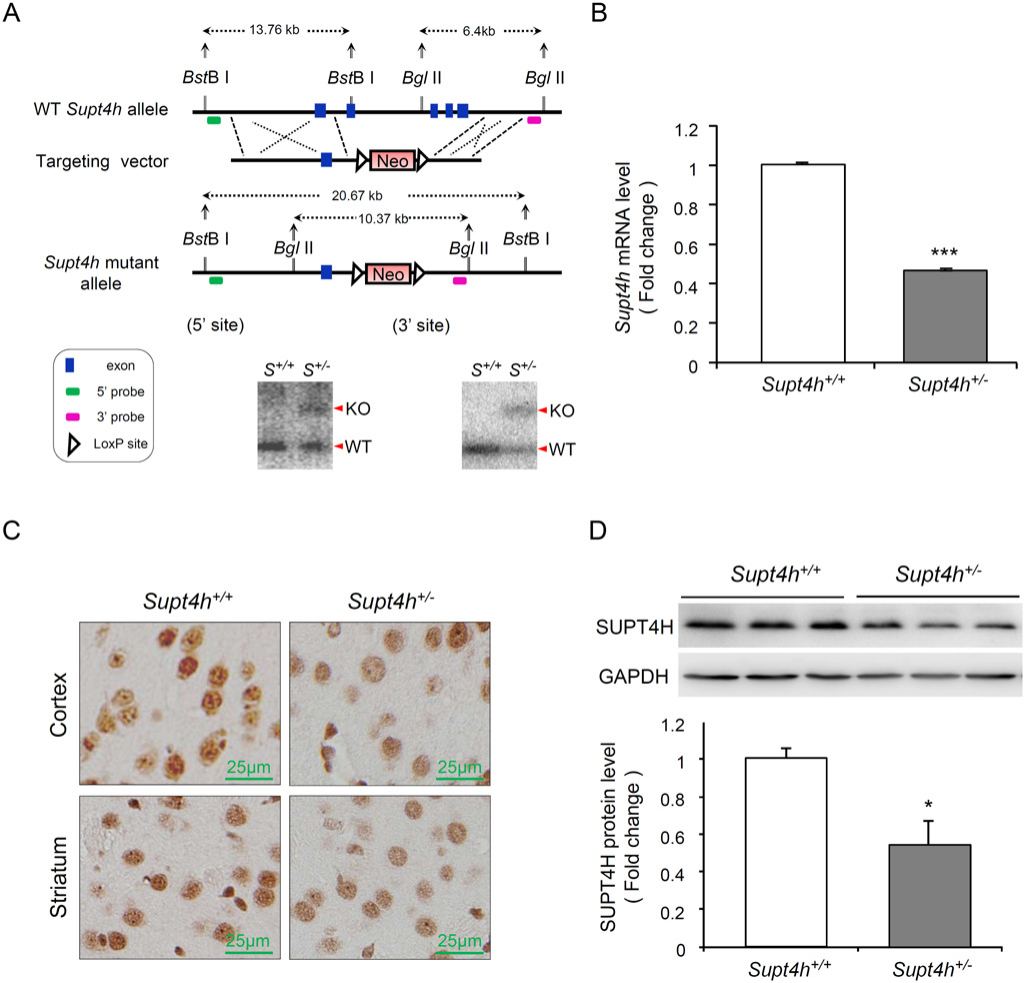
Creation and characterization of *Supt4h* knockout mice. (A) Genomic organization of the mouse *Supt4h* locus (Top) and structure of the targeting vector (Middle). In the allele carrying the *Supt4h* deletion, a neo cassette specifying resistance to the antibiotic G418 in animal cells replaced the DNA fragment encompassing exon 2 to exon 5 of *Supt4h* via homologous recombination (Bottom). Positions of 5’ and 3’ flanking probes used in Southern blot analysis, and predicted sizes of restriction fragments detected by these probes are shown. Genomic DNA of C57BL6/129 mice (*S* ^*+/+*^) and their *Supt4h* ^*+/-*^ (*S* ^*+/-*^) littermates was subjected to Southern blot analysis using the 5’ and 3’ probes separately. (B) *Supt4h* mRNA levels were assessed by qRT-PCR using the brain tissue of *Supt4h* ^*+/+*^ and *Supt4h* ^*+/-*^ mice. The abundance in *Supt4h* ^*+/+*^ mice was set as 1, after normalization with *U6* RNA. (C) SUPT4H protein level in the striatum and cortex of indicated mice was analyzed by immunohistochemistry (IHC) using antibody against SUPT4H. (D) Protein lysates collected from the cerebrum of indicated mice were analyzed by Western blot using anti-SUPT4H antibody. GAPDH served as loading control. Data are presented as the mean ± SEM (n = 3 in each group; *, *p* < 0.05; ***, *p* <0.001 by Student’s *t*-test). The mice were sacrificed at the age of 12 weeks for analyses.

To learn about the effects of more widespread and prolonged reduction of *Supt4h* expression in mice, and also to determine the effects of such reduction on phenotypes characteristic of HD, we first constructed a C57BL6/129-derived mouse strain deleted for *Supt4h* using conventional genetic knockout approaches (Fig 2). We obtained mice having a deletion of one *Supt4h* allele, as confirmed by Southern blot analysis (Fig 2A); however, mating of such *Supt4h* ^*+/-*^ animals failed to generate viable offspring having deletions in both *Supt4h* alleles. Instead, analysis of embryos indicated that homozygous knockout of *Supt4h* was associated with embryonic lethality at day E7.5 (S1 Table).

Using quantitative RT-PCR (qRT-PCR) to assess *Supt4h* mRNA abundance in *Supt4h* ^*+/-*^ mice, we found that *Supt4h* transcripts in cerebral tissue lysates were decreased to approximately 50% of the abundance observed in *Supt4h* ^*+/+*^ littermates (Fig 2B); consistent with this observation, SUPT4H protein was reduced in the striatal and cortical regions of the brain, as determined by immunohistochemistry staining (Fig 2C) and Western blot analysis (Fig 2D). Mice showing this extent of decrease in SUPT4H abundance, which corresponds to the decrease that results in reduced mutant HTT toxicity in cultured striatal neurons [13], were maintained for 18 months without apparent effects on lifespan or motor function.

### Biochemical effects of deletion of one *Supt4h* allele in R6/2 mice

**Fig 3:**
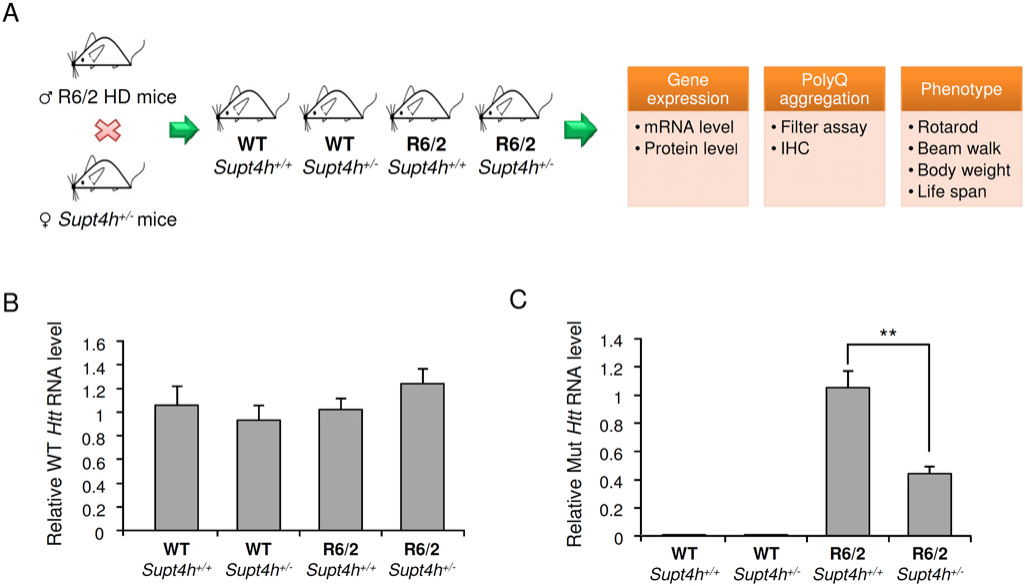
Effect of heterozygous deletion of *Supt4h* on expression of mutant and wild-type *Htt* alleles in R6/2 mice. (A) Outline of procedures used to generate heterozygous deletion of *Supt4h* in R6/2 HD mice by crossing with *Supt4h* ^*+/-*^ mice, followed by summary of biochemical and phenotypic analyses of their offspring. (B) Expression of wild-type murine *Htt* gene was assessed by qRT-PCR using *U6* as an internal control. Samples were collected from left cerebrum of indicated animals at the age of 12 weeks, and the gene expression in WT mice containing two functional *Supt4h* alleles was set as 1. (C) Same as (B), except that expression of mutant *Htt* allele was analyzed and mutant *Htt* expression in R6/2 mice containing two functional *Supt4h* alleles was set as 1. Data are presented as the mean ± SEM (n = 3 in each group; **, *p* <0.01 by Student’s *t*-test).

R6/2 mice, which carry a transgenically introduced first exon of human *HTT* containing an expanded CAG repeat and which robustly show biochemical and behavior characteristics of HD [20,21], have been used extensively to evaluate events that may affect humans afflicted with HD. To evaluate the effects of perturbed *Supt4h* expression in these mice, we generated a line of R6/2-derived *Supt4h* ^*+/-*^ animals (Fig 3A). As was observed for *Supt4h* ^*+/-*^ mice in the C57BL6/129 strain background, whole brains collected from R6/2 *Supt4h* ^*+/-*^ animals showed approximately 50% reduction of *Supt4h* abundance relative to R6/2 *Supt4h* ^*+/+*^ animals (Fig 2). Quantitative RT-PCR using conditions that distinguish between expression of wild-type and mutant *Htt* alleles indicated that deletion of one *Supt4h* allele in R6/2 mice was accompanied by a marked reduction in mutant *Htt* mRNA in brain tissue, whereas mRNA production by the wild-type *Htt* allele was unaltered by the *Supt4h* gene deletion (Fig 3B, 3C). Western blotting using an antibody that detects only the mutant form of HTT confirmed that expression of the mutant *Htt* allele was reduced in zQ175 mice treated with ASO directed against either *Supt4h* or *Htt*; however, ASO against *Htt* also reduces protein produced by the normal *Htt* allele, while ASO directed against *Supt4h* did not (S2 Fig). In R6/2 mouse experiments, slot blot assays and antibody that detects only the mutant form confirmed the ability of a null mutation in one *Supt4h* allele to reduce expression of mutant HTT in *Supt4h* knockout mice as shown in Fig 3B, 3C.

**Fig 4:**
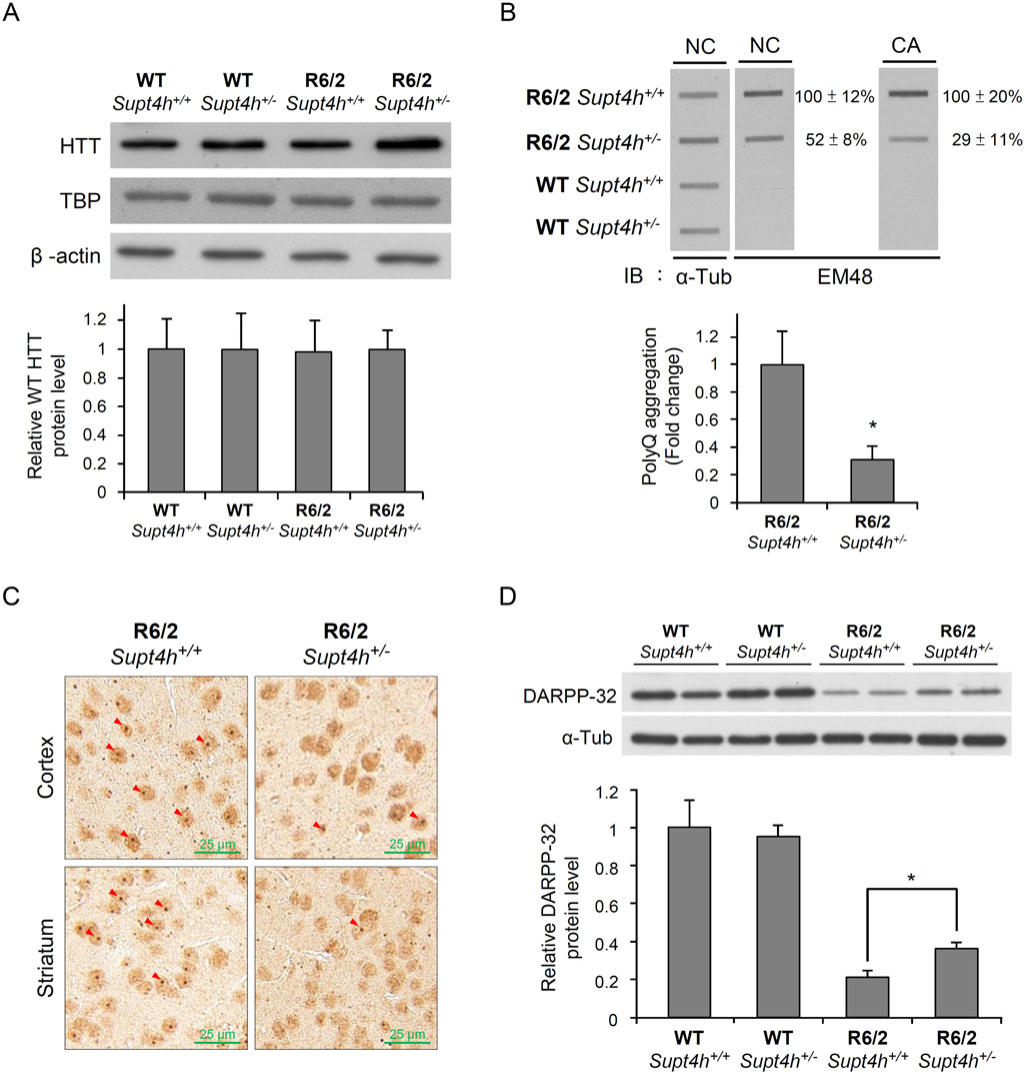
Mutant HTT aggregation in the brain of R6/2 mice deleted for one *Supt4h* allele. (A) Wild-type HTT protein levels were examined by Western blot analysis using brain lysates collected from right cerebrum of animals as described in Materials and Methods and in Fig 3B. TATA-binding protein (TBP) produced by a gene containing 13–15 consecutive CAA/CAG repeats was also analyzed. β-actin served as loading control. (B) Brain lysates collected from 12-week-old mice were loaded onto a cellulose acetate CA membrane, which traps only aggregated protein. Mutant HTT protein was detected using EM48 antibody. Nitrocellulose (NC) membranes were employed for slot blot assays to determine protein abundance; α-Tubulin served as a loading control. The values shown are means ± SEM, and the relative protein aggregation in tissues of R6/2 HD mice having two or one allele of *Supt4h* is presented in the bottom panel. (C) Representative IHC images of cerebral tissue of 12-week-old R6/2 (HD) mice having either one or two alleles of *Supt4h* are shown. HTT aggregates were detected using an antibody against ubiquitin, which is recruited to and co-localized with aggregates in the brain of HD mice [54]. The positions of aggregates are indicated by arrowheads. (D) DARPP-32 protein abundance was analyzed by Western blot analysis using brain lysates collected from R6/2 mice at the age of 12 weeks either intact in the *Supt4h* locus or deleted for one *Supt4h* allele. The level of WT mice having two *Supt4h* alleles was set to 1, after normalization with α-Tubulin. Data are presented as the mean ± SEM (n = 3 in each group; *, *p* < 0.05 by Student’s *t*-test).

Aggregation of mutant HTT is a prominent feature of HD during disease progression, and reduction of such aggregation has been reported to rescue neurons from dysfunction and cell death [22–24]. Our earlier studies using cultured cells showed that both the production and aggregation of mutant HTT is decreased by siRNA directed against *Supt4h* [13]. We observed that R6/2 mice deleted for one *Supt4h* allele showed a similarly reduced abundance of mutant HTT protein and a decrease in HTT protein aggregates (Fig 4B, 4C, and S3 Fig), while showing no change in the amount of normal HTT protein synthesized from a coding sequence containing a short TNR (Fig 4A). Additionally, reduction of the DARPP-32 protein, which is highly enriched in medium-sized spiny neurons and has been reported to be down-regulated concurrently with early neuronal dysfunction in the R6/2 mouse model of HD [25,26], was partially reversed in mouse brains by deletion of one *Supt4h* allele (Fig 4D).

### Reduction of *Supt4h* suppresses motor decline and prolongs survival of R6/2 mice

**Fig 5:**
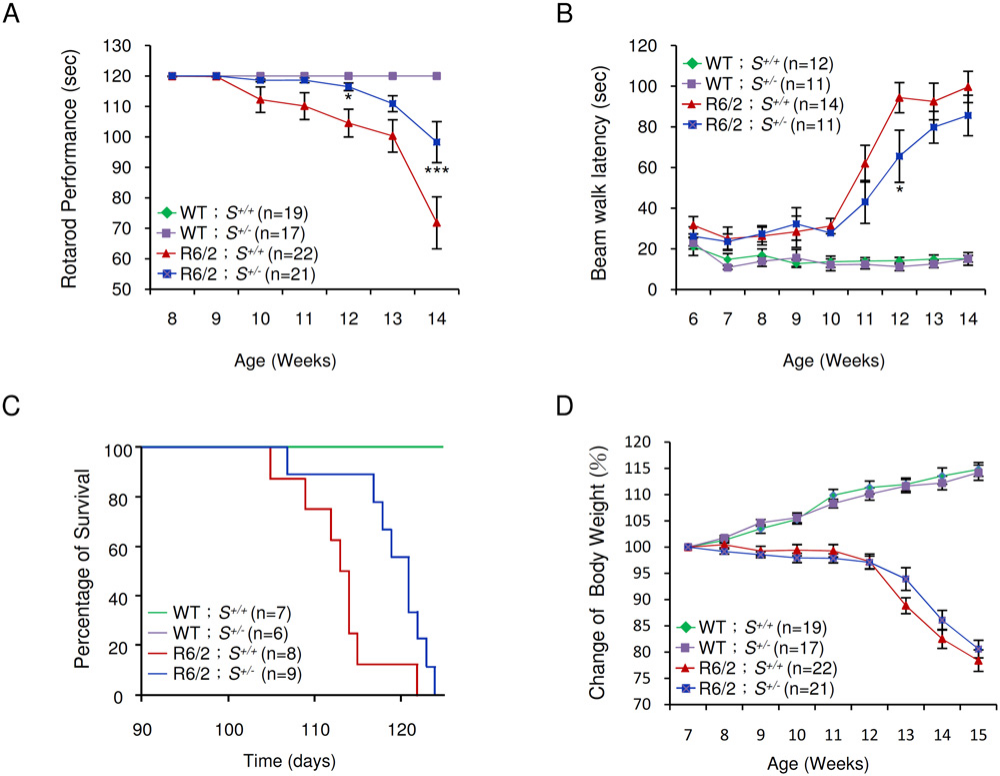
Effect of *Supt4h* deletion on motor function and lifespan of R6/2 mice. (A) Mice at the age of 8 to 14 weeks were tested for rotarod performance as described in Materials and Methods. (B) Latency of indicated animals, at the age of 6 to 14 weeks, on beam walking was analyzed. Data are presented as the mean ± SEM. *, *p* < 0.05; ***, *p* < 0.001 in comparison with R6/2 *S* ^+/+^ mice, using two-way ANOVA followed by Bonferroni *post hoc* test. (C) Longevity of indicated animals was recorded. Compared to R6/2 *S* ^+/+^ mice, HD animals with a single allele of *Supt4h* deletion (R6/2 *S* ^+/-^) showed a longer lifespan (*p* = 0.0204, Log-rank test). (D) Body weight was measured weekly and its change relative to the body weight at the age of 7 weeks is shown. Results were collected from mice at 7 through 15 weeks of age. The number of mice (n) used in each individual assay is indicated.

Typically, R6/2 mice show severe impairment of motor coordination by 8–12 weeks of age [27], and die between 13 and 16 weeks of age [16]. The progressive deterioration in motor function can be detected by reduction in the length of time that the mice can remain on a rotating rod—the so-called “rotarod assay” [27]. We employed rotarod performance assays to compare the motor function of R6/2 mice deleted in one *Supt4h* allele with that of R6/2 *Supt4h* ^+/+^ littermates. As observed previously [28] R6/2 *Supt4h* ^*+/+*^ mice showed a progressive decline in motor function starting at 10 weeks of age; however, in R6/2 *Supt4h* ^*+/-*^ mice, the decline was not apparent until 13 weeks of age (Fig 5A)—suggesting that reduction of SUPT4H abundance by half in these animals, as indicated above, is sufficient to yield measurable benefits in motor function. Similarly, R6/2 mice having one *Supt4h* allele deleted showed better performance in a beam walking test (Fig 5B) commonly used as another parameter of motor function in the HD mouse model system [27,28]. R6/2 mice carrying a heterozygous deletion in *Supt4h* also showed a longer lifespan (Fig 5C) than did *Supt4h* ^*+/+*^ animals; however, no detectable effect of an *Supt4h* deletion on the loss of body weight that is characteristic of HD progression in R6/2 mice (Fig 5D) was observed.

## Discussion

The results reported here demonstrate that experimentally induced decrease of the transcription elongation protein SUPT4H in brain and spinal cord tissues of murine models of Huntington’s disease results in selectively decreased expression of mutant huntingtin alleles, and that these events are associated with reduction of HTT protein aggregates, delay in the impairment of motor function seen in R6/2 HD mice as the animals age, and an increase in the R6/2 mouse lifespan.

SUPT4H and its yeast counterpart, Spt4, function in cells by binding to Spt5/SUPT5H to form a protein complex; the N-terminal region of SUPT5H then interacts with the C-terminal region of RNA polymerase II (Pol II), an event that is thought to tighten the Pol II clamp around DNA templates and limit dissociation of Pol II from DNA during transcription pauses [13,29–31]. Data obtained by crystallographic analyses indicate that SUPT4H and Spt4 do not directly contact the polymerase [32–35] and in yeast, null mutations of *spt4*, unlike those of *spt5* do not preclude cell viability [36]. Our earlier findings that dissociation of the Pol II complex from DNA in template segments containing expanded TNRs is increased during Spt4 deficiency, and that interference with the function of Spt4 or SUPT4H decreases expression of genes containing expanded TNR regions in cultured cells—while not significantly affecting transcription of genes containing shorter TNRs or no TNRs at all [13]—have raised the prospect that targeting the function of SUPT4H may be a useful strategy for treatment of HD and possibly other TNR diseases. The murine results reported here support this notion.

The potential therapeutic value of reducing HTT expression in the brains of individuals afflicted with HD or other polyQ disorders is well recognized [37], and antisense oligonucleotides that target *Htt* sequences common to mutant and normal alleles have been shown to reduce overall production of *Htt* mRNA and protein in brain tissue when delivered into the cerebrospinal fluid of HD-afflicted mice by transient infusion [18]. Such non-selective decrease in expression of both *Htt* alleles prevented the appearance of HD-disease symptom, and did not result in observable detrimental effects during the duration of those experiments. However, bi-allelic *Htt* inactivation in the forebrain and testes leads to progressive neuronal degeneration and sterility [38], and selective targeting expression from mutant *HTT* alleles has been a desirable therapeutic objective. Selective knockdown of mutant *HTT* mRNA translation recently has been reported using single-stranded RNA (ssRNA) that target expanded CAG repeat segments within *HTT* transcripts [39]; while this approach reduces mutant HTT protein, it does not affect the abundance of mutant *HTT* mRNA, which can also contribute to cellular toxicity [40–42]. siRNA and ASOs that target polymorphic gene sequences that differ in mutant and normal *HTT* alleles have also been reported to achieve allele-specific inhibition [43–46]. However, downregulation of mutant *HTT* mRNA by the targeting of *SUPT4H* is a strategy that is independent of fortuitously occurring sequence differences in mutant and wild-type *HTT* alleles and additionally may also be applicable toward the treatment of other disorders caused by TNR expansions.

Even in the presence of SUPT4H, mRNA produced by an *Htt* allele containing expanded TNRs is less abundant than mRNA from a co-existing allele having unexpanded TNRs [13,19,47]. The expanded polyQ protein encoded by the mutant allele is correspondingly less abundant [13,19], although the upregulation of translation mediated by increased binding of the MID-1 protein to expanded CAG repeats can elevate mutant HTT protein above the level of wild-type HTT [47]. Reduction of SUPT4H by half in the brains of zQ175 mice by intracerebroventricular bolus injection of ASO or in R6/2 mice by deletion of one *Supt4h* allele results in a decrease in mutant *Htt* transcription beyond the already reduced level of mRNA production. In the R6/2 strain, which displays phenotypic features seen in human HD, such reduction resulting from deletion of a single *Supt4h* allele was associated with partial reversal of the HD-like phenotypic properties. The non-HD mouse strain having an *Supt4h* allele deleted showed no overt functional impairment during an 18-month period of observation.

Our results show that knockdown of *Supt4h* in murine tissues to 30–50% of normal does not preclude survival of mice after birth. However, notwithstanding the viability of yeast carrying null mutations in the *Supt4h* ortholog *SPT4* [13], and the minimal effect of shRNA knockdown of *Supt4h* on RNA-SEQ profiles in mice [13], the lethality we observed for *Supt4h* ^-/-^ embryos argues that one or more actions of SUPT4H may be required for adequate transcription during embryogenesis of one or more of multiple normal mouse genes that contain >40 trinucleotide repeats.

Mutant alleles in most HD patients do not contain repeats of the length necessary to yield HD-related phenotypes in transgenic mice [48,49]; and additionally, the consequences of *Supt4h* knockdown potentially may be affected by genetic variation in the native cellular abundance of SUPT4H (and perhaps of SUPT5H its transcription elongation complex partner) in brain tissues of different individuals. While the investigations reported here indicate that the allele-specific effects of *Supt4h* knockdown reported previously in cultured cells occur also in mouse HD model systems and that reduction of *Supt4h* expression can result in disease-related consequences in a mouse HD model, the parameters that affect selective expression of mutant vs. wild type *HTT* alleles require further investigation before the clinical relevance of our findings can be established.

## Materials and Methods

### Ethics statement

All animal experiments were performed in accordance with the guidelines established by the Institutional Animal Care and Use Committee (IACUC) of Academia Sinica. All the experimental protocols were approved by IACUC and the approval number is 11–12–253. Mice were sacrificed by CO_2_ inhalation according to the approved protocol for tissue collection and IHC analysis.

### Animals

The zQ175 mouse strain, which carries a normal murine *Htt* allele and a knock-in (KI) mutant *Htt/HTT mouse/human hybrid* allele containing around 188 CAG repeats [15], was provided by the CHDI Foundation, Inc. Male R6/2 [B6CBA-Tg (HDexon1) 62Gpb/1J] [16] mice, which contain an N-terminally-truncated mutant *HTT* allele containing a long CAG repeat, were obtained from Jackson Laboratories (Bar Harbor, ME, USA) and mated to normal females of mouse strain B6CBAFI/J. The genotype of offspring was verified by polymerase chain reaction (PCR), using genomic DNA extracted from tail tips and primers that specifically target the mutant *Htt* transgene. The number of CAG repeats of R6/2 mice used in this study was 240 ± 10 (mean ± SEM). *Supt4h* knockout was generated in a C57BL6/129S6 hybrid mouse line background using a conventional gene targeting approach. The colony was maintained by breeding *Supt4h* ^*+/-*^ males with C57BL6 females. PCR genotyping was carried out using primer sets Supt4h WT and Supt4h MT to detect intact and genetically deleted alleles of *Supt4h* respectively. The nucleotide sequence of these primers is shown in S2 Table.

To produce R6/2 mice that contain or lack a deletion of one *Supt4h* allele, R6/2 males were crossed with *Supt4h* ^*+/-*^ females, and progeny were subjected to genotyping for both *Supt4h* intact and genetically deleted alleles and for the R6/2 human *Htt* transgene containing an expanded CAG repeat. The biochemical and behavioral experiments were performed using littermates from the same population. Mice were housed at the Institute of Biomedical Sciences Animal Care Facility (Taipei, Taiwan) under a 12h light-dark cycle. All procedures were accomplished using a protocol approved by the Academia Sinica Institutional Animal Care and Utilization Committee (Taipei, Taiwan).

### Anti-sense oligonucleotide (ASO) treatment

The Supt4h ASO (5’-CGACACTTGTGTCCCCTGCT-3’) used in this study was a 20-mer oligonucleotide that contains a phosphorothioate backbone and a chimeric 2’-*O*-methoxyethyl (MOE)/DNA design [50] containing five MOE-modified nucleotides at each end of a centered stretch of ten DNAs. Oligonucleotide was synthesized [17] and solubilized in PBS. zQ175 KI mice [15] were kindly provided by CHDI and received ASO (300 μg) or PBS via intracerebroventricular (ICV) bolus injections at the age of 5.5 months. Tissues were collected 4 weeks after a single ICV bolus injection, and RNA or protein was extracted as previously described [17].

### RNA isolation and qRT-PCR

Total RNA was extracted from isolated tissues using Trizol reagent (Invitrogen) and the abundance of *Supt4h* and *Htt* transcripts was assessed by quantitative real time RT-PCR (qRT-PCR) as described previously [13]. For samples collected from zQ175 HD mice, 1 μg of total RNA was converted to cDNA, followed by qRT-PCR analysis using ABI StepOnePlus Real-Time PCR System (Life Technologies). In zQ175 HD mice, the KI mutant allele contains a DNA fragment of human *HTT*, which is distinct from murine *Htt* in nucleotide sequence. PCR primers were designed to correspond to species-specific sequences and thus to differentiate mRNA produced from the wild-type vs. KI mutant allele. Samples collected from R6/2 experiments were analyzed as described above, except that 3 μg of total RNA was used for the synthesis of cDNA, and qRT-PCR was performed using ABI PRISM 7500 Sequence Detection System (Life Technologies). Relative gene expression was determined by the 2^-△△Ct^ method after normalization with either *U6* or 18S ribosomal RNA. Oligonucleotides used for qPCR are summarized and shown in S2 Table.

### Southern blot analysis

Genomic DNA extracted from the tails of mice was digested by restriction enzymes *Bgl* II or *Bst*B l (New England BioLabs). DNA was then electrophoresed on agarose gels, transferred to Hybond-N^+^ nylon membranes (GE Healthcare), and fixed on membranes using UV cross-linker (UV Stratalinker 1800, Stratagene), as previously described [51]. DNA probes for detection of the *Supt4h* locus were generated by PCR and labeled with ^32^P using Amersham Rediprime II DNA Labeling System (GE Healthcare). After hybridization with Supt4h 5’- or 3’-probe in Church buffer (0.25 M sodium phosphate, 1 mM EDTA, 1% BSA, 7% SDS, and 10 mg/ml salmon sperm DNA) overnight, the membrane was rinsed twice with buffer I (2X SSC, 0.1% SDS) at 30°C for 30 minutes, followed by buffer II (0.2X SSC, 0.1% SDS) at 60°C. DNA fragments recognized by the probes were monitored by Typhoon 9410 Variable Mode Imager (GE Healthcare).

### Preparation of anti-SUPT4H antibody

Plasmid construct pPAL7-HA-Supt4h, which expresses full-length murine SUPT4H, was created by PCR amplification of a DNA fragment encoding SUPT4H and the HA-epitope and subsequently sub-cloning of this PCR product in *E*. *coli* on expression vector pPAL7. The expression construct was introduced into *E*. *coli* BL21 (DE3), and production of SUPT4H protein was induced by isopropyl-β-D-thiogalactopyranoside (IPTG, Promega) as per the manufacturer’s protocol.

For protein purification, BL21 cells were lysed using a Microfluidizer (Microfluidics Corp.) in buffer A (0.1 M sodium phosphate, pH 7.2). After centrifugation, the supernatant was mixed with 1 ml Profinity eXact purification resin (Bio-Rad) and incubated at room temperature for 2 hours. Reaction mixtures were loaded onto a Poly-Prep Chromatography Column (Bio-Rad), washed with 10 column volumes of wash buffer, and incubated in 2 column volumes of elution buffer (100 mM sodium phosphate, 100 mM sodium fluoride, pH 7.2) at 4°C overnight. The purified protein was subsequently eluted, transferred to a dialysis membrane (Cellu‧Sep T1, Uptima), and sent to LTK BioLaboratories for immunization of rabbits. Antibody against SUPT4H was validated by Western blotting using purified recombinant protein, protein lysates of mammalian 293T cells expressing ectopic SUPT4H, and brain lysates obtained from C57BL6 mice.

### Western blot analysis

Tissues collected from zQ175 HD mice were homogenized by dounce homogenizer using cell lysis buffer (Cell Signaling). For R6/2 or *Supt4h* genetically modified mice, brain lysates were prepared similarly using lysis buffer (10 mM HEPES, 1 mM DTT, 200 μM Na_3_VO_4_, 8.5% (w/v) sucrose, protease inhibitor). Immunoblotting was performed as previously described [13]. In brief, equal amounts of protein were resolved by electrophoresis on 8, 12, or 15% sodium dodecyl sulfate (SDS)-polyacrylamide gels, transferred onto immobilon-P polyvinylidene difluoride (PVDF) membranes (Millipore), and probed with anti-SUPT4H, anti-α-Tubulin (DM1A, Sigma), anti-TBP (T1827, Sigma), anti-HTT (MAB2166, Chemicon), anti-polyQ (clone 5TF1–1C2, MAB1574, Chemicon), anti-β-actin (GTX109639, GeneTex), anti-GAPDH (GTX100118, GeneTex), or anti-DARPP-32 (#2302, Cell Signaling) antibodies. After incubation with a horseradish peroxidase (HRP)-conjugated secondary antibody for 1 h, the immunoreactive signals were detected by ECL reagent (enhanced chemiluminescence, PerkinElmer).

### Filter-retardation assay

Filter-retardation assays were performed as previously described [13]. Briefly, brain lysates collected from R6/2-derived animals were loaded through a slot-blot manifold (Bio-Rad) onto CA membranes (cellulose acetate, 0.2 μm pore size; Schleicher & Schuell), which retain SDS-insoluble protein aggregates. Membranes were blocked with 5% skim milk in TBST (10 mM Tris-HCl, pH 8.0, 150 mM NaCl, 0.05% Tween 20) and probed with EM48 antibody (MAB5374, Chemicon) at 4°C overnight. EM48 antibody identifies N-terminal huntingtin fragments containing a long stretch of polyglutamine, and is particularly efficient for detecting human huntingtin aggregates, whereas the antibody has only weak affinity for rodent HTT protein [52]. After incubation with the corresponding secondary antibody, immunoreactive signals were detected by ECL reagent and recorded using Fuji X-ray film.

### Immunohistochemistry

Animals were anesthetized before perfusion with 4% paraformaldehyde in PBS (pH 7.4). Brains were removed, post-fixed with 4% paraformaldehyde at 4°C overnight, and embedded in paraffin as previously described [53]. Serial coronal sections (5 μm) were deparaffinized by xylene substitute (Fluka) and rehydrated by serial alcohol dilution and subsequent PBS rinse. After heating and cooling in retrieval solution (pH 6.0, DakoCytomation), brain sections were permeabilized by 0.5% Triton X-100 and blocked with 10% goat serum for 1 h. Sections were then stained with primary antibodies against SUPT4H or ubiquitin (DakoCytomation) at 4°C overnight, followed by incubation with the corresponding secondary antibody for 1 h. To enhance the signal, Vectastain ABC kit (Vector Laboratories) was applied before staining with diaminobenzidine (DakoCytomation). Nuclei were stained with hematoxylin or methyl green.

### Phenotype assays

Body weights of mice were recorded weekly. Motor coordination was assessed using a rotarod apparatus (MK-660D, Muromachi-Kikai) retaining at a constant speed (12 rpm) over a period of 2 minutes, as previously described [28]. Animals were pre-trained at the age of 7 weeks to become acquainted with the apparatus. Then mice were tested three times per week from the age of 8 to 14 weeks.

Beam walk analysis was applied to assess motor coordination [28]. Mice were trained to traverse a circular beam having a diameter of 17 mm, followed by testing on an 11-mm-diameter beam once per week. Results were recorded as the duration of time (Latency) spent by mice to walk across the 80-cm-long beam. Latency was recorded as 120 seconds when mice spent more than 120 seconds traversing the beam.

### Statistical analysis

Values shown in the figures are presented as mean ± SEM. All statistical analyses were carried out by Student’s *t*-test except indicated otherwise. Rotarod performance, beam walk test, and change of body weight were analyzed using two-way analysis of variance (ANOVA), followed by a post-hoc Bonferroni multiple comparison test. Survival statistics were performed using Log-rank test. All tests were performed using the SigmaPlot software, version 10.0. A value of p<0.05 was considered statistically significant.

## Supporting Information

S1 Fig Mutant, but not wild-type *Htt* gene expression is down-regulated by Supt4h ASO in the cortex of zQ175 HD mice. zQ175 HD mice were treated with Supt4h ASO as described in Fig 1, except that the cortex was collected for further analyses. (A) *Supt4h* mRNA was assessed by quantitative RT-PCR. The mRNA level of PBS-treated samples (mock) was set to 1, and relative *Supt4h* mRNA abundance in ASO-treated specimen is shown. (B) Wild-type (WT) and mutant (Mut) allele expression of *Htt* was assessed by qRT-PCR in mock and Supt4h ASO-treated samples. The abundance of *Htt* mRNA produced from WT allele in mock samples was set to 1. Htt ASO that targets against wild-type (WT) and mutant (Mut) *Htt* non-selectively was included as a control (*, *p* <0.05; **, *p* <0.01 by Student’s *t* test).
(TIF)

S2 Fig Mutant HTT is selectively reduced by Supt4h ASO in zQ175 HD mice. Protein lysates collected from ASO-treated mice (n = 3) as described in Fig 1 were analyzed by Western blot using MAB2166 antibody that detects both wild-type (WT) and mutant (Mut) HTT proteins, and MAB1574 that only probes the mutant one. The positions of Mut and WT HTT are indicated by arrowheads. The bands were scanned and quantified using Multi Gauge (www.lifescience.fujifilm.com) and the ratios of Mut to WT HTT protein are shown for each sample. α-Tubulin served as a loading control. Mean values and ± SDs are indicated. Lysates collected from the cerebrum of B6CBAFI/J mice were also included as a control to validate the specificity of MAB1574 antibody.
(TIF)

S3 Fig R6/2 mice deleted for one *Supt4h* allele show a decrease of HTT protein aggregates. 12-week-old R6/2 (HD) mice having either one or two alleles of *Supt4h* were subjected to IHC staining. HTT aggregates were detected using an anti-mHTT antibody EM48. The positions of aggregates are indicated by arrowheads.
(TIF)

S1 Table Embryonic lethality of *Supt4h* knockout mice. (TIF)

S2 Table Nucleotide sequence of primers used in this study. (TIF)

## References

[1] Ashley CT Jr., Warren ST (1995) Trinucleotide repeat expansion and human disease. Annu Rev Genet 29: 703–728. 8825491 10.1146/annurev.ge.29.120195.003415

[2] Orr HT, Zoghbi HY (2007) Trinucleotide repeat disorders. Annu Rev Neurosci 30: 575–621. 17417937 10.1146/annurev.neuro.29.051605.113042

[3] Lopez Castel A, Cleary JD, Pearson CE (2010) Repeat instability as the basis for human diseases and as a potential target for therapy. Nat Rev Mol Cell Biol 11: 165–170. 10.1038/nrm2854 20177394

[4] Finkbeiner S (2011) Huntington's Disease. Cold Spring Harb Perspect Biol 3. 10.1101/cshperspect.a007476 PMC3098678 21441583

[5] Group. HsDCR (1993) A novel gene containing a trinucleotide repeat that is expanded and unstable on Huntington's disease chromosomes. The Huntington's Disease Collaborative Research Group. Cell 72: 971–983. 8458085 10.1016/0092-8674(93)90585-e

[6] Ross CA (2002) Polyglutamine pathogenesis: emergence of unifying mechanisms for Huntington's disease and related disorders. Neuron 35: 819–822. 12372277 10.1016/s0896-6273(02)00872-3

[7] Zoghbi HY, Orr HT (2000) Glutamine repeats and neurodegeneration. Annu Rev Neurosci 23: 217–247. 10845064 10.1146/annurev.neuro.23.1.217

[8] Gatchel JR, Zoghbi HY (2005) Diseases of unstable repeat expansion: mechanisms and common principles. Nat Rev Genet 6: 743–755. 16205714 10.1038/nrg1691

[9] Cummings CJ, Zoghbi HY (2000) Fourteen and counting: unraveling trinucleotide repeat diseases. Hum Mol Genet 9: 909–916. 10767314 10.1093/hmg/9.6.909

[10] Kumari D, Lokanga R, Yudkin D, Zhao XN, Usdin K (2012) Chromatin changes in the development and pathology of the Fragile X-associated disorders and Friedreich ataxia. Biochim Biophys Acta 1819: 802–810. 10.1016/j.bbagrm.2011.12.009 22245581 PMC3370136

[11] Meola G, Cardani R (2014) Myotonic dystrophies: An update on clinical aspects, genetic, pathology, and molecular pathomechanisms. Biochim Biophys Acta. 10.1016/j.bbadis.2014.05.019 24882752

[12] Usdin K, Hayward BE, Kumari D, Lokanga RA, Sciascia N, et al (2014) Repeat-mediated genetic and epigenetic changes at the FMR1 locus in the Fragile X-related disorders. Front Genet 5: 226 10.3389/fgene.2014.00226 25101111 PMC4101883

[13] Liu CR, Chang CR, Chern Y, Wang TH, Hsieh WC, et al (2012) Spt4 is selectively required for transcription of extended trinucleotide repeats. Cell 148: 690–701. 10.1016/j.cell.2011.12.032 22341442

[14] Heikkinen T, Lehtimaki K, Vartiainen N, Puolivali J, Hendricks SJ, et al (2012) Characterization of neurophysiological and behavioral changes, MRI brain volumetry and 1H MRS in zQ175 knock-in mouse model of Huntington's disease. PLoS One 7: e50717 10.1371/journal.pone.0050717 23284644 PMC3527436

[15] Menalled LB, Kudwa AE, Miller S, Fitzpatrick J, Watson-Johnson J, et al (2012) Comprehensive behavioral and molecular characterization of a new knock-in mouse model of Huntington's disease: zQ175. PLoS One 7: e49838 10.1371/journal.pone.0049838 23284626 PMC3527464

[16] Mangiarini L, Sathasivam K, Seller M, Cozens B, Harper A, et al (1996) Exon 1 of the HD gene with an expanded CAG repeat is sufficient to cause a progressive neurological phenotype in transgenic mice. Cell 87: 493–506. 8898202 10.1016/s0092-8674(00)81369-0

[17] Rigo F, Chun SJ, Norris DA, Hung G, Lee S, et al (2014) Pharmacology of a central nervous system delivered 2'-O-methoxyethyl-modified survival of motor neuron splicing oligonucleotide in mice and nonhuman primates. J Pharmacol Exp Ther 350: 46–55. 10.1124/jpet.113.212407 24784568 PMC4056267

[18] Kordasiewicz HB, Stanek LM, Wancewicz EV, Mazur C, McAlonis MM, et al (2012) Sustained therapeutic reversal of Huntington's disease by transient repression of huntingtin synthesis. Neuron 74: 1031–1044. 10.1016/j.neuron.2012.05.009 22726834 PMC3383626

[19] Hu J, Matsui M, Gagnon KT, Schwartz JC, Gabillet S, et al (2009) Allele-specific silencing of mutant huntingtin and ataxin-3 genes by targeting expanded CAG repeats in mRNAs. Nat Biotechnol 27: 478–484. 10.1038/nbt.1539 19412185 PMC2765218

[20] Murphy KP, Carter RJ, Lione LA, Mangiarini L, Mahal A, et al (2000) Abnormal synaptic plasticity and impaired spatial cognition in mice transgenic for exon 1 of the human Huntington's disease mutation. J Neurosci 20: 5115–5123. 10864968 10.1523/JNEUROSCI.20-13-05115.2000 PMC6772265

[21] Meade CA, Deng YP, Fusco FR, Del Mar N, Hersch S, et al (2002) Cellular localization and development of neuronal intranuclear inclusions in striatal and cortical neurons in R6/2 transgenic mice. J Comp Neurol 449: 241–269. 12115678 10.1002/cne.10295

[22] Ravikumar B, Vacher C, Berger Z, Davies JE, Luo S, et al (2004) Inhibition of mTOR induces autophagy and reduces toxicity of polyglutamine expansions in fly and mouse models of Huntington disease. Nat Genet 36: 585–595. 15146184 10.1038/ng1362

[23] Wolfgang WJ, Miller TW, Webster JM, Huston JS, Thompson LM, et al (2005) Suppression of Huntington's disease pathology in Drosophila by human single-chain Fv antibodies. Proc Natl Acad Sci U S A 102: 11563–11568. 16061794 10.1073/pnas.0505321102 PMC1183604

[24] Chiang MC, Chen HM, Lai HL, Chen HW, Chou SY, et al (2009) The A2A adenosine receptor rescues the urea cycle deficiency of Huntington's disease by enhancing the activity of the ubiquitin-proteasome system. Hum Mol Genet 18: 2929–2942. 10.1093/hmg/ddp230 19443488

[25] Bibb JA, Yan Z, Svenningsson P, Snyder GL, Pieribone VA, et al (2000) Severe deficiencies in dopamine signaling in presymptomatic Huntington's disease mice. Proc Natl Acad Sci U S A 97: 6809–6814. 10829080 10.1073/pnas.120166397 PMC18747

[26] Jin K, LaFevre-Bernt M, Sun Y, Chen S, Gafni J, et al (2005) FGF-2 promotes neurogenesis and neuroprotection and prolongs survival in a transgenic mouse model of Huntington's disease. Proc Natl Acad Sci U S A 102: 18189–18194. 16326808 10.1073/pnas.0506375102 PMC1312383

[27] Carter RJ, Lione LA, Humby T, Mangiarini L, Mahal A, et al (1999) Characterization of progressive motor deficits in mice transgenic for the human Huntington's disease mutation. J Neurosci 19: 3248–3257. 10191337 10.1523/JNEUROSCI.19-08-03248.1999 PMC6782264

[28] Hsiao HY, Chiu FL, Chen CM, Wu YR, Chen HM, et al (2014) Inhibition of soluble tumor necrosis factor is therapeutic in Huntington's disease. Hum Mol Genet 23: 4328–4344. 10.1093/hmg/ddu151 24698979

[29] Parsons MA, Sinden RR, Izban MG (1998) Transcriptional properties of RNA polymerase II within triplet repeat-containing DNA from the human myotonic dystrophy and fragile X loci. J Biol Chem 273: 26998–27008. 9756950 10.1074/jbc.273.41.26998

[30] Gnatt AL, Cramer P, Fu J, Bushnell DA, Kornberg RD (2001) Structural basis of transcription: an RNA polymerase II elongation complex at 3.3 A resolution. Science 292: 1876–1882. 11313499 10.1126/science.1059495

[31] Salinas-Rios V, Belotserkovskii BP, Hanawalt PC (2011) DNA slip-outs cause RNA polymerase II arrest in vitro: potential implications for genetic instability. Nucleic Acids Res 39: 7444–7454. 10.1093/nar/gkr429 21666257 PMC3177194

[32] Guo M, Xu F, Yamada J, Egelhofer T, Gao Y, et al (2008) Core structure of the yeast spt4-spt5 complex: a conserved module for regulation of transcription elongation. Structure 16: 1649–1658. 10.1016/j.str.2008.08.013 19000817 PMC2743916

[33] Wenzel S, Martins BM, Rosch P, Wohrl BM (2010) Crystal structure of the human transcription elongation factor DSIF hSpt4 subunit in complex with the hSpt5 dimerization interface. Biochem J 425: 373–380. 10.1042/BJ20091422 19860741

[34] Klein BJ, Bose D, Baker KJ, Yusoff ZM, Zhang X, et al (2011) RNA polymerase and transcription elongation factor Spt4/5 complex structure. Proc Natl Acad Sci U S A 108: 546–550. 10.1073/pnas.1013828108 21187417 PMC3021056

[35] Martinez-Rucobo FW, Sainsbury S, Cheung AC, Cramer P (2011) Architecture of the RNA polymerase-Spt4/5 complex and basis of universal transcription processivity. EMBO J 30: 1302–1310. 10.1038/emboj.2011.64 21386817 PMC3094117

[36] Malone EA, Fassler JS, Winston F (1993) Molecular and genetic characterization of SPT4, a gene important for transcription initiation in Saccharomyces cerevisiae. Mol Gen Genet 237: 449–459. 8483459 10.1007/BF00279450

[37] Zuccato C, Valenza M, Cattaneo E (2010) Molecular mechanisms and potential therapeutical targets in Huntington's disease. Physiol Rev 90: 905–981. 10.1152/physrev.00041.2009 20664076

[38] Dragatsis I, Levine MS, Zeitlin S (2000) Inactivation of Hdh in the brain and testis results in progressive neurodegeneration and sterility in mice. Nat Genet 26: 300–306. 11062468 10.1038/81593

[39] Yu D, Pendergraff H, Liu J, Kordasiewicz HB, Cleveland DW, et al (2012) Single-stranded RNAs use RNAi to potently and allele-selectively inhibit mutant huntingtin expression. Cell 150: 895–908. 10.1016/j.cell.2012.08.002 22939619 PMC3444165

[40] Yuan Y, Compton SA, Sobczak K, Stenberg MG, Thornton CA, et al (2007) Muscleblind-like 1 interacts with RNA hairpins in splicing target and pathogenic RNAs. Nucleic Acids Res 35: 5474–5486. 17702765 10.1093/nar/gkm601 PMC2018611

[41] Mykowska A, Sobczak K, Wojciechowska M, Kozlowski P, Krzyzosiak WJ (2011) CAG repeats mimic CUG repeats in the misregulation of alternative splicing. Nucleic Acids Res 39: 8938–8951. 10.1093/nar/gkr608 21795378 PMC3203611

[42] Krzyzosiak WJ, Sobczak K, Wojciechowska M, Fiszer A, Mykowska A, et al (2012) Triplet repeat RNA structure and its role as pathogenic agent and therapeutic target. Nucleic Acids Res 40: 11–26. 10.1093/nar/gkr729 21908410 PMC3245940

[43] van Bilsen PH, Jaspers L, Lombardi MS, Odekerken JC, Burright EN, et al (2008) Identification and allele-specific silencing of the mutant huntingtin allele in Huntington's disease patient-derived fibroblasts. Hum Gene Ther 19: 710–719. 10.1089/hum.2007.116 18549309

[44] Pfister EL, Kennington L, Straubhaar J, Wagh S, Liu W, et al (2009) Five siRNAs targeting three SNPs may provide therapy for three-quarters of Huntington's disease patients. Curr Biol 19: 774–778. 10.1016/j.cub.2009.03.030 19361997 PMC2746439

[45] Zhang Y, Engelman J, Friedlander RM (2009) Allele-specific silencing of mutant Huntington's disease gene. J Neurochem 108: 82–90. 10.1111/j.1471-4159.2008.05734.x 19094060 PMC3166352

[46] Southwell AL, Skotte NH, Kordasiewicz HB, Ostergaard ME, Watt AT, et al (2014) In vivo evaluation of candidate allele-specific mutant huntingtin gene silencing antisense oligonucleotides. Mol Ther. 10.1038/mt.2014.153 PMC4429695 25101598

[47] Krauss S, Griesche N, Jastrzebska E, Chen C, Rutschow D, et al (2013) Translation of HTT mRNA with expanded CAG repeats is regulated by the MID1-PP2A protein complex. Nat Commun 4: 1511 10.1038/ncomms2514 23443539

[48] Lee CY, Cantle JP, Yang XW (2013) Genetic manipulations of mutant huntingtin in mice: new insights into Huntington's disease pathogenesis. FEBS J 280: 4382–4394. 10.1111/febs.12418 23829302 PMC3770892

[49] Menalled LB, Chesselet MF (2002) Mouse models of Huntington's disease. Trends Pharmacol Sci 23: 32–39. 11804649 10.1016/s0165-6147(00)01884-8

[50] DeVos SL, Miller TM (2013) Antisense oligonucleotides: treating neurodegeneration at the level of RNA. Neurotherapeutics 10: 486–497. 10.1007/s13311-013-0194-5 23686823 PMC3701770

[51] Cheng TH, Gartenberg MR (2000) Yeast heterochromatin is a dynamic structure that requires silencers continuously. Genes Dev 14: 452–463. 10691737 PMC316382

[52] Li H, Li SH, Cheng AL, Mangiarini L, Bates GP, et al (1999) Ultrastructural localization and progressive formation of neuropil aggregates in Huntington's disease transgenic mice. Hum Mol Genet 8: 1227–1236. 10369868 10.1093/hmg/8.7.1227

[53] Di Pardo A, Amico E, Favellato M, Castrataro R, Fucile S, et al (2014) FTY720 (fingolimod) is a neuroprotective and disease-modifying agent in cellular and mouse models of Huntington disease. Hum Mol Genet 23: 2251–2265. 10.1093/hmg/ddt615 24301680

[54] Chiang MC, Chen CM, Lee MR, Chen HW, Chen HM, et al (2010) Modulation of energy deficiency in Huntington's disease via activation of the peroxisome proliferator-activated receptor gamma. Hum Mol Genet 19: 4043–4058. 10.1093/hmg/ddq322 20668093

