# Supplementary figures and images for: Effects on Murine Behavior and Lifespan of Selectively Decreasing Expression of Mutant Huntingtin Allele by Supt4h Knockdown
Source: PLoS Genet. 2015 Mar 11;11(3):e1005043. doi: 10.1371/journal.pgen.1005043 (PMC4356588; doi:10.1371/journal.pgen.1005043)

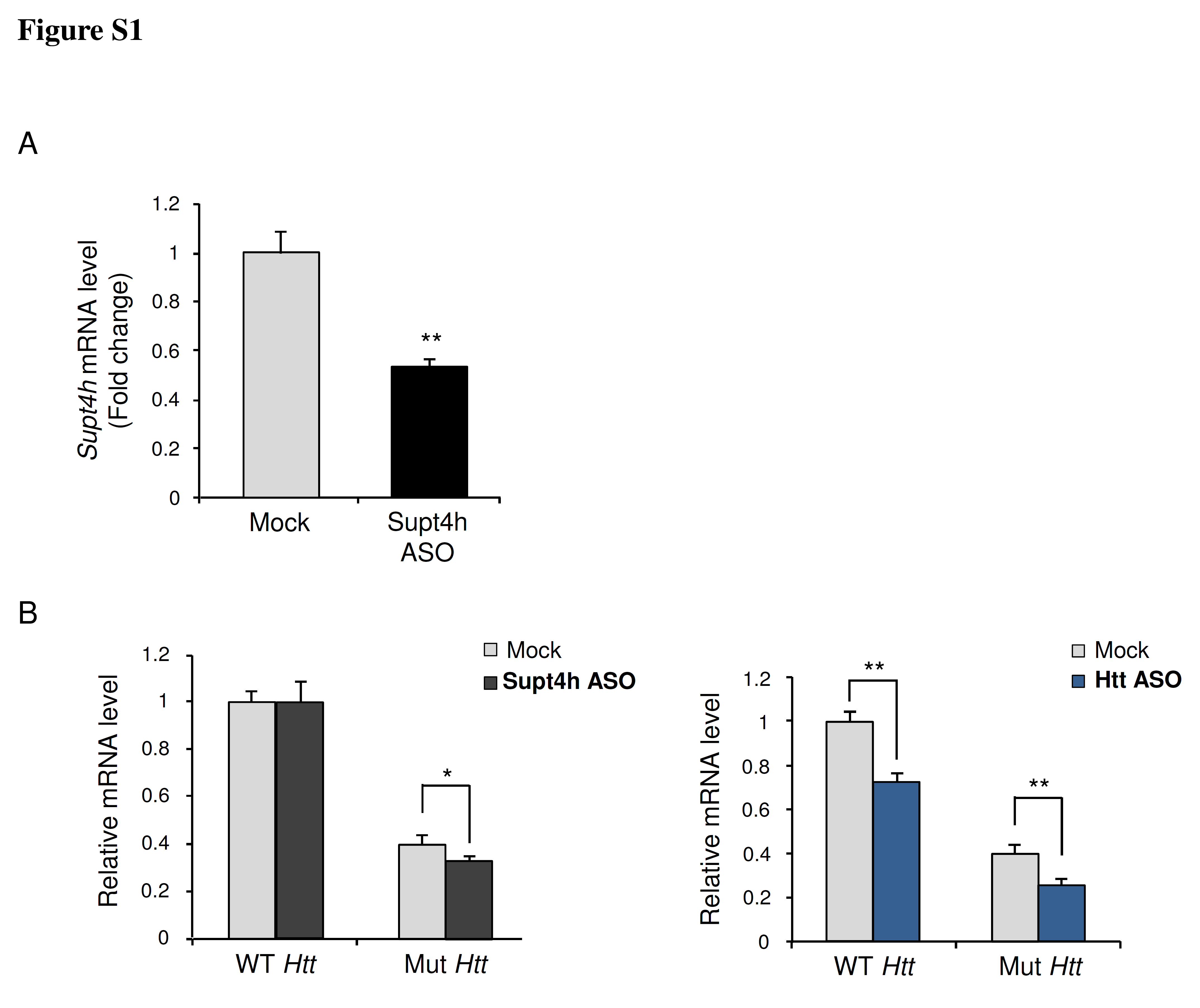

Supplement: S1 Fig — zQ175 HD mice were treated with Supt4h ASO as described in Fig 1, except that the cortex was collected for further analyses. (A) Supt4h mRNA was assessed by quantitative RT-PCR. The mRNA level of PBS-treated samples (mock) was set to 1, and relative Supt4h mRNA abundance in ASO-treated specimen is shown. (B) Wild-type (WT) and mutant (Mut) allele expression of Htt was assessed by qRT-PCR in mock and Supt4h ASO-treated samples. The abundance of Htt mRNA produced from WT allele in mock samples was set to 1. Htt ASO that targets against wild-type (WT) and mutant (Mut) Htt non-selectively was included as a control (*, p <0.05; **, p <0.01 by Student’s t test). (TIF) [file pgen.1005043.s001.tif]

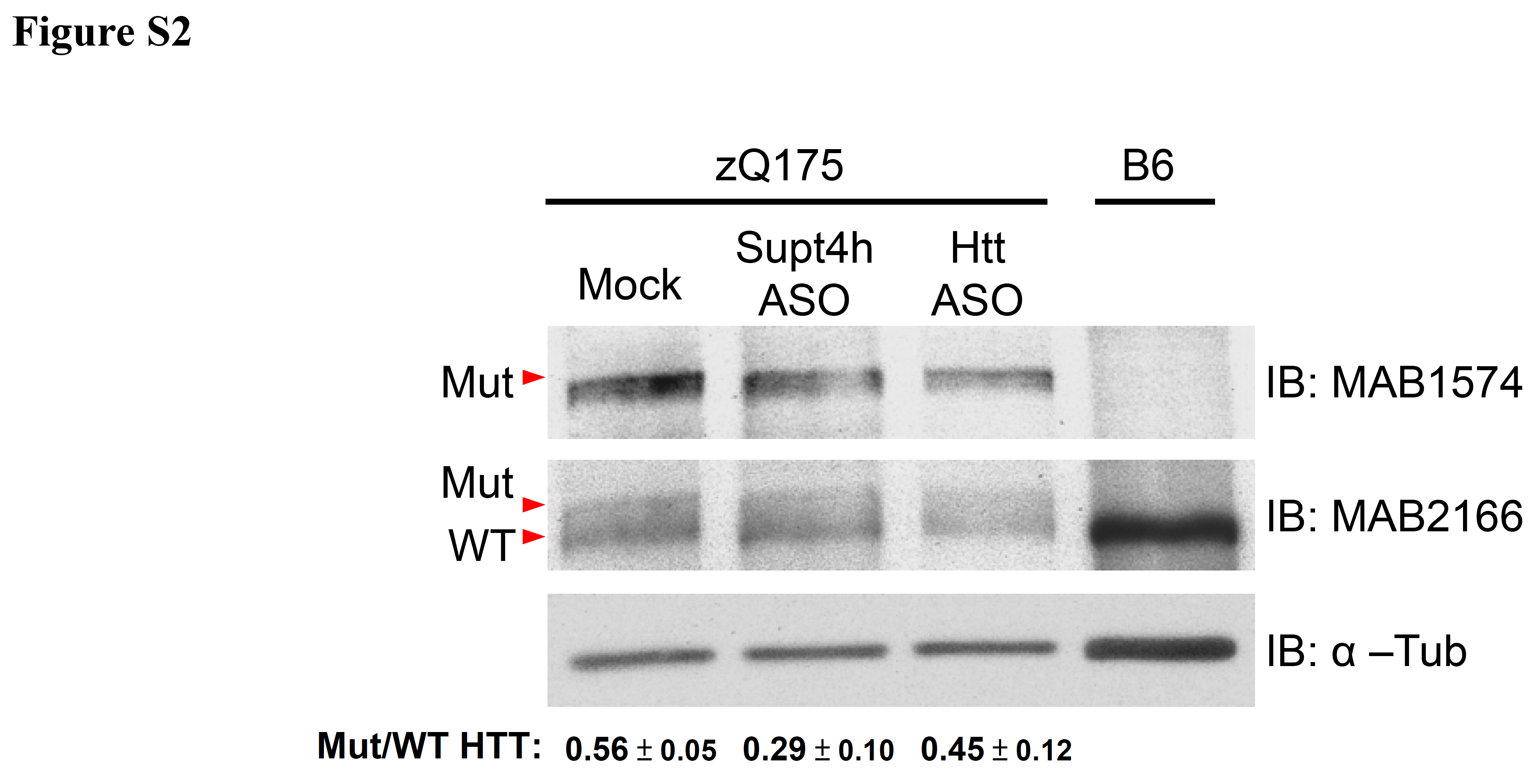

Supplement: S2 Fig — Protein lysates collected from ASO-treated mice (n = 3) as described in Fig 1 were analyzed by Western blot using MAB2166 antibody that detects both wild-type (WT) and mutant (Mut) HTT proteins, and MAB1574 that only probes the mutant one. The positions of Mut and WT HTT are indicated by arrowheads. The bands were scanned and quantified using Multi Gauge (www.lifescience.fujifilm.com) and the ratios of Mut to WT HTT protein are shown for each sample. α-Tubulin served as a loading control. Mean values and ± SDs are indicated. Lysates collected from the cerebrum of B6CBAFI/J mice were also included as a control to validate the specificity of MAB1574 antibody. (TIF) [file pgen.1005043.s002.tif]

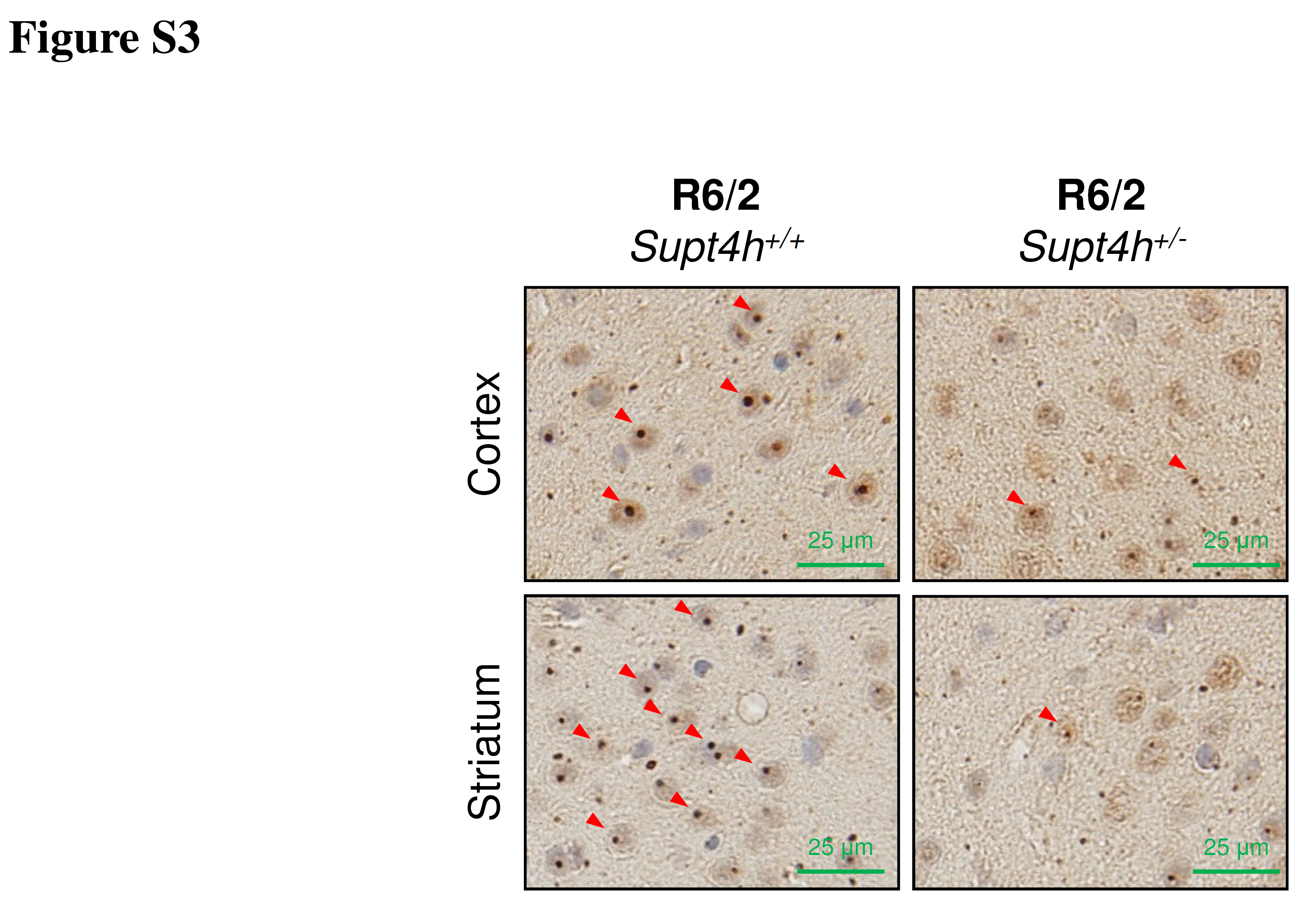

Supplement: S3 Fig — 12-week-old R6/2 (HD) mice having either one or two alleles of Supt4h were subjected to IHC staining. HTT aggregates were detected using an anti-mHTT antibody EM48. The positions of aggregates are indicated by arrowheads. (TIF) [file pgen.1005043.s003.tif]

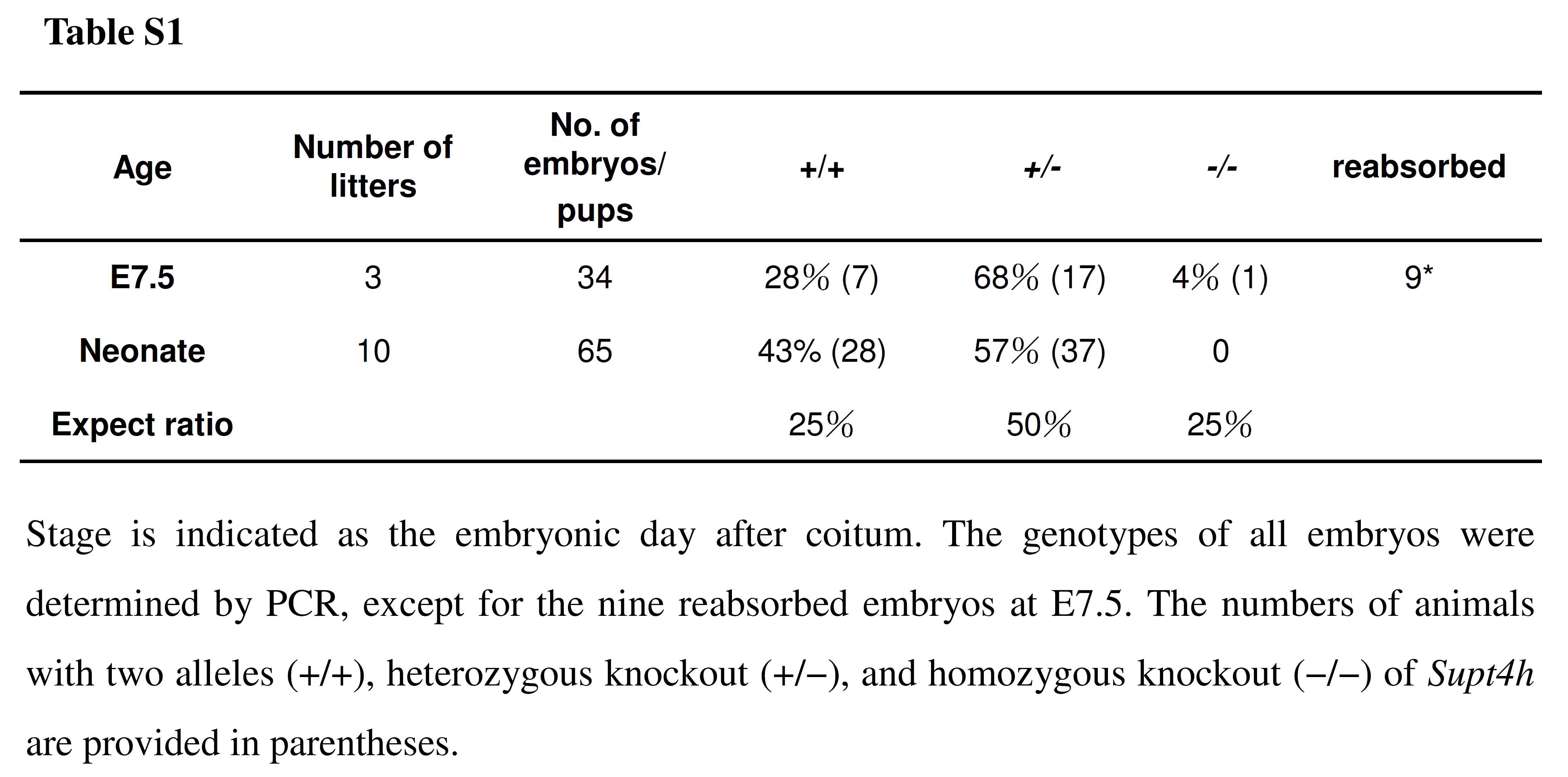

Supplement: S1 Table — (TIF) [file pgen.1005043.s004.tif]

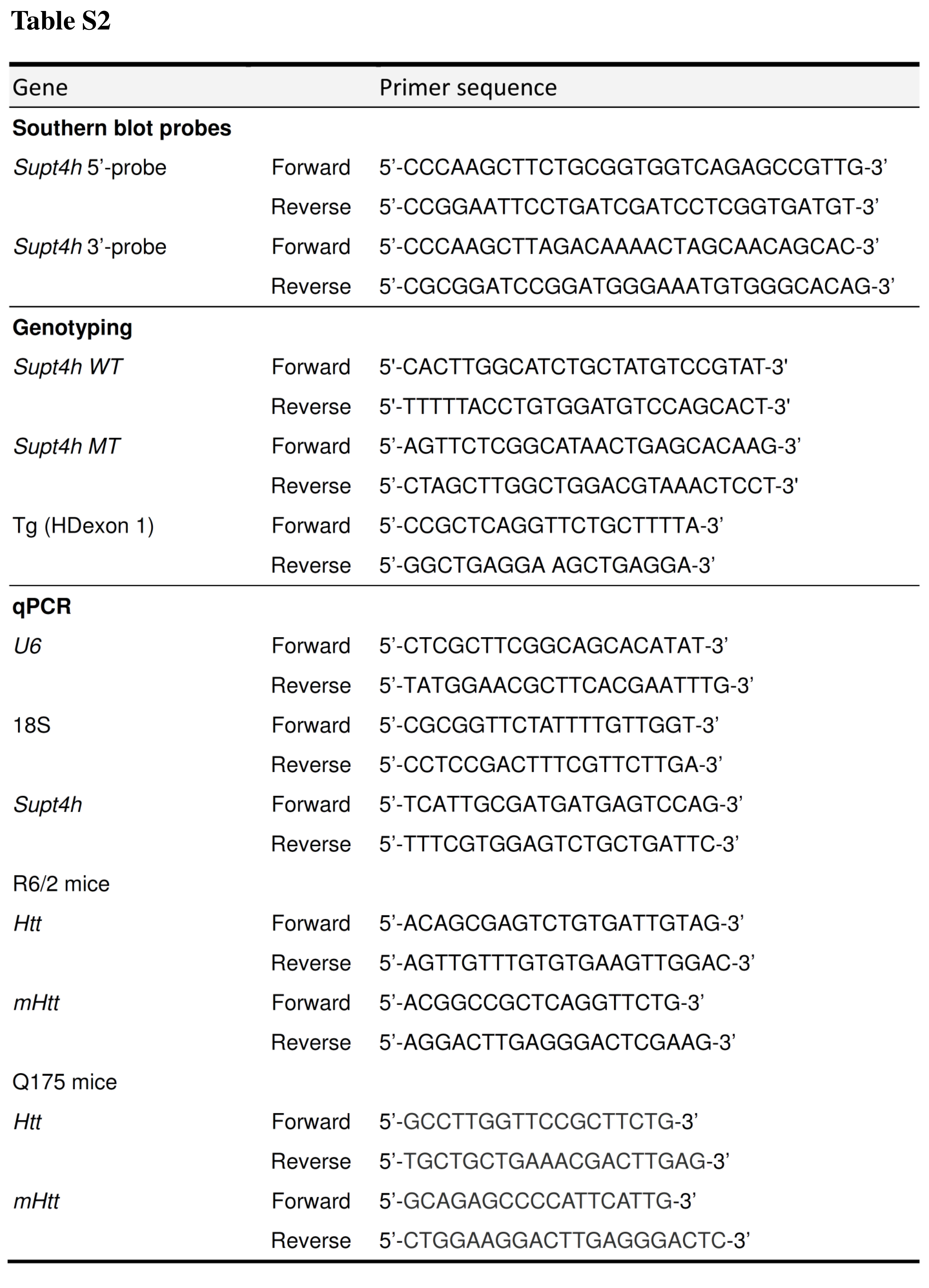

Supplement: S2 Table — (TIF) [file pgen.1005043.s005.tif]
